# Factors Driving Background Choice in Scorpionfish

**DOI:** 10.1002/ece3.71876

**Published:** 2025-07-28

**Authors:** Leonie John, Matteo Santon, Nico K. Michiels

**Affiliations:** ^1^ Animal Evolutionary Ecology, Institute of Evolution and Ecology University of Tübingen Tübingen Germany; ^2^ Ecology of Vision Group, School of Biological Sciences University of Bristol Bristol UK; ^3^ Department of Biology University of Padova Padova Italy

**Keywords:** background matching, camouflage, disruptive colouration, heterogeneous habitat, visual modelling

## Abstract

For a successful hunt, marine ambush predators such as scorpionfish need to be well camouflaged to deceive their prey. When the natural environment is heterogeneous, one strategy to maintain camouflage is choosing backgrounds to achieve better crypsis. We tested if two Mediterranean scorpionfish species, *Scorpaena maderensis* and *S. porcus* , select backgrounds according to this strategy. By using visual modelling on data extracted from standardised photography of natural substrates, we show that scorpionfish should be able to visually distinguish between different substrates using achromatic rather than chromatic contrast information. We then conducted a behavioural experiment where scorpionfish could choose between backgrounds that were similar or different (lighter and darker) to their own average body luminance, as measured in previous studies. Unexpectedly, scorpionfish did not choose backgrounds of luminance similar to their own but instead settled preferentially on darker backgrounds. To investigate these results further, we characterised the colouration of scorpionfish's distinct pattern components after their choice using calibrated image analysis. We show that the darker parts of the fish pattern best matched the dark background for both species, and that *S. porcus* increased internal pattern contrast when choosing the darker background. We therefore propose that the preference for dark backgrounds enhances camouflage via disruptive colouration. The choice for specific backgrounds, in addition to their ability to rapidly change colour and intensify disruptive colouration, can potentially allow scorpionfish to camouflage in a broad range of microhabitats.

## Graphical abstract

Scorpionfishes are well camouflaged ambush predators. We use behavioural tests in combination with standardised photography and visual modelling to quantify camouflage parameters. We discuss the relevance of chromatic and achromatic cues in the fishes' natural environment for background choice and the potential use of different camouflage strategies, such as disruptive colouration.

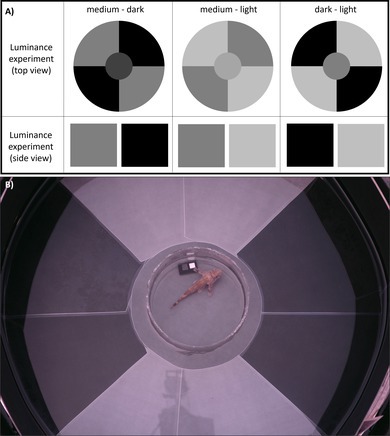

## 1 Introduction

Animals use different strategies to camouflage in heterogeneous or changing environments (Hughes et al. 2019). They can flexibly change colour (Duarte et al. 2017; John et al. 2023, 2024), have a generalist body colouration that allows camouflage on a diversity of backgrounds (Briolat et al. 2021; Nokelainen et al. 2019), actively choose backgrounds that maximise crypsis (Dyer and Stevens 2024; Green et al. 2024; Stevens and Ruxton 2019; Twort and Stevens 2023), or show combinations of these. In line with the latter strategy, some marine fishes have already been shown to preferentially settle on backgrounds that match their own body colouration or pattern (Smithers et al. 2018; Tyrie et al. 2015). For example, rock pool gobies preferred substrates on which they better matched the background (Smithers et al. 2018). This study considered overall body colouration rather than specific parts of the fish pattern to assess background matching (Smithers et al. 2017, 2018). However, some fishes might have distinctive pattern components. When settling on a given background, camouflage can be achieved with different strategies. For background matching, the overall colouration or pattern of the fish should be similar to that of the background (Stevens and Merilaita 2009a). On the other hand, disruptive colouration works by maximising internal contrast and achieving differential blending of certain pattern parts with the background, that is, partial background matching (Stevens and Merilaita 2009b). This strategy works by disrupting a fish's outline and therefore delays detection or recognition by the observer (Stevens and Merilaita 2009b).

Scorpionfishes (family Scorpaenidae) are well‐camouflaged sit‐and‐wait marine benthic predators that ambush approaching prey by sudden suction feeding. The Mediterranean species *Scorpaena maderensis* (Valenciennes, 1833) and *Scorpaena porcus* (Linnaeus, 1758) are found on a variety of substrates that differ in colour, luminance and pattern. Both species are known to rapidly change their appearance depending on background features. When placed on differently coloured uniform backgrounds, they change luminance (calculated from the prey's perspective) and to some extent also colour (John et al. 2023). Luminance changes also regulate internal contrast and average patch size of the skin pattern. While the dominant pattern of both species seems to be fixed, rapid changes in luminance of different bars within the pattern can mediate internal pattern contrast and could therefore promote disruptive colouration on contrast‐rich backgrounds (John et al. 2024). Mediterranean *Scorpaena* are dichromats, with peak cone sensitivity at short (455 nm) and medium (530 nm) wavelengths (Govardovskii and Zueva 1988; Schweikert et al. 2018). As such, they have limited ability to distinguish between differently coloured substrates, especially if characterised mostly by green, reds and browns, such as the Mediterranean benthos. If changes in body colouration are mediated by vision (Duarte et al. 2017; Stevens 2016), it is therefore possible that scorpionfish adjustment to different backgrounds is not primarily driven by chromatic but achromatic vision.

In this study, we investigated (1) how well scorpionfish should be able to distinguish different natural substrates. Here, we used calibrated photography and visual modelling to quantify chromatic and achromatic contrast between natural substrate types. We expected low chromatic contrast but higher achromatic contrast between the substrate types because of the scorpionfish's dichromatic vision.

We further investigated (2) whether scorpionfish use perceived substrate luminance as a cue to actively choose backgrounds to settle on. To test this, we conducted a behavioural experiment where scorpionfish were allowed to choose between grey backgrounds of different luminance. We expected scorpionfish to prefer backgrounds that were closer to their average body luminance, according to the strategy of background matching.

Because background matching is not the only possible strategy to achieve camouflage, we also explored (3) whether scorpionfish background choice could be explained by disruptive colouration. To assess this, after scorpionfish settled on their chosen background, we documented the colouration of their distinct pattern components. We then modelled, from the visual perspective of a prey fish, the perceived luminance of the different pattern components of scorpionfish colouration and their achromatic contrast against the chosen background and against each other as a measure of internal pattern contrast.

## 2 Methods

The study was carried out at the Station de Recherches Sous‐marines et Océanographiques (STARESO), Corsica, France, in June and July 2023. The Madeira Rockfish *S. maderensis* and the Black Scorpionfish *S. porcus* are benthic ambush predators that are frequent in the area. Both species can change body colouration (John et al. 2023) and pattern contrast (John et al. 2024) in response to their background and likely also use other camouflage strategies (Santon et al. 2018).

### 2.1 Visual Contrasts of Natural Backgrounds

We took standardised photos of natural substrates where scorpionfish were caught for the behavioural experiment, and where these species are known to occur from previous studies (John et al. 2023, 2024). We focused on six common substrate types: (1) *rubble and sand*, consisting of rubble covered with sand and epiphytes, (2) *seagrass leaves*, partially covered by epiphytes, (3) *seagrass stems*, located just above the plant's roots, (4) *turf algae*, (5) *yellow algae* (*Dictyota* cf. *fasciola*), and (6) a species of *red sponge* (cf. *Crambe crambe*). Standardised raw photos were taken while SCUBA‐diving at 6 m depth using a calibrated Nikon D4 DLSR camera (Nikkor 60 mm macro lens). This was an intermediate depth of the range where we typically caught scorpionfish (2–10 m). We used a pole with a dark grey standard (9% grey) attached to its tip to place the standards in the images perpendicularly to the lens of the camera. The pole also served to standardise the distance between substrate and camera in each picture (~50 cm).

To analyze substrate colour, we used the Multispectral Image Calibration and Analysis (MICA) Toolbox plugin (version 2.2.2) (van den Berg et al. 2020; Troscianko and Stevens 2015) for ImageJ (version 1.54d). Images were normalised with the 9% grey standard and converted into 32‐bit multispectral images. For each image, we defined one or more regions of interest (ROIs), selecting only areas in the photo that were in the same plane as the grey standard and had the same exposure. Measurements of multiple ROIs were averaged for one image. All images were then batch‐processed using a custom‐written script for the MICA toolbox in ImageJ (John et al. 2023). Normalised images were converted to cone catches using the spectral sensitivity of the camera and of the modelled observer, and for the spectra used during photography and the model illuminant, we used light measurements taken at our sampling location at 6 m depth (Santon et al. 2018). We modelled the vision of *Scorpaena porcus* (cone sensitivities peaking at 455 and 530 nm [Govardovskii and Zueva 1988; Schweikert et al. 2018]). The spectral sensitivity of *S. maderensis* is not known, so we assumed it to be similar to that of *S. porcus* . We used the Receptor Noise Limited model (Vorobyev and Osorio 1998) in the R (version 4.1.1) (R Core Team 2021) package pavo (Maia et al. 2019) to calculate chromatic and achromatic contrast between each substrate type, using average cone catch measures for each image. We assumed a Weber fraction of 0.05 for the cones and for the luminance channel (defined as the long wavelength cone [John et al. 2023; Lythgoe 1979]) (Champ et al. 2016; Olsson et al. 2018) and a cone ratio of 1:1 (Lyall 1957). Contrasts are reported as Just Noticeable Differences (JND), where values below one JND indicate an indistinguishable contrast under optimal viewing conditions, and values above one indicate an increased probability of detection (Siddiqi et al. 2004; Vorobyev and Osorio 1998). Three JND are often considered as a more conservative approach to interpret results because a detection threshold of one JND is unlikely under natural viewing conditions (Abernathy et al. 2017; Siddiqi et al. 2004; da Silva et al. 2020; Stevens et al. 2014).

### 2.2 Background Choice

#### 2.2.1 Experimental Setup and Procedure

In the choice experiment, we tested whether scorpionfish prefer a background with a perceived luminance that is more similar to their average body luminance, compared to a darker and lighter background. We restricted the testing to backgrounds varying in luminance because scorpionfish change luminance more than colour (John et al. 2023), and because natural substrates differ more in perceived luminance than in colour (see Section 3.1). Scorpionfish for the behavioural experiment were caught under the station's general sampling permit, using hand nets while SCUBA diving at depths of 2 to 10 m. We tested 23 *S. maderensis* and 27 *S. porcus* , and after the observations all individuals were eventually returned to the field. We followed the EU animal welfare legislation's directive (Directive 2010/63/EU) to ensure that our research was not likely to cause pain, suffering, distress, or lasting harm equivalent to, or higher than, that caused by the introduction of a needle in accordance with good veterinary practice. Before the experiment, scorpionfish were kept in shaded outside flow‐through seawater tanks (210 × 120 × 50 cm^3^) exposed to natural light.

**FIGURE 1:**
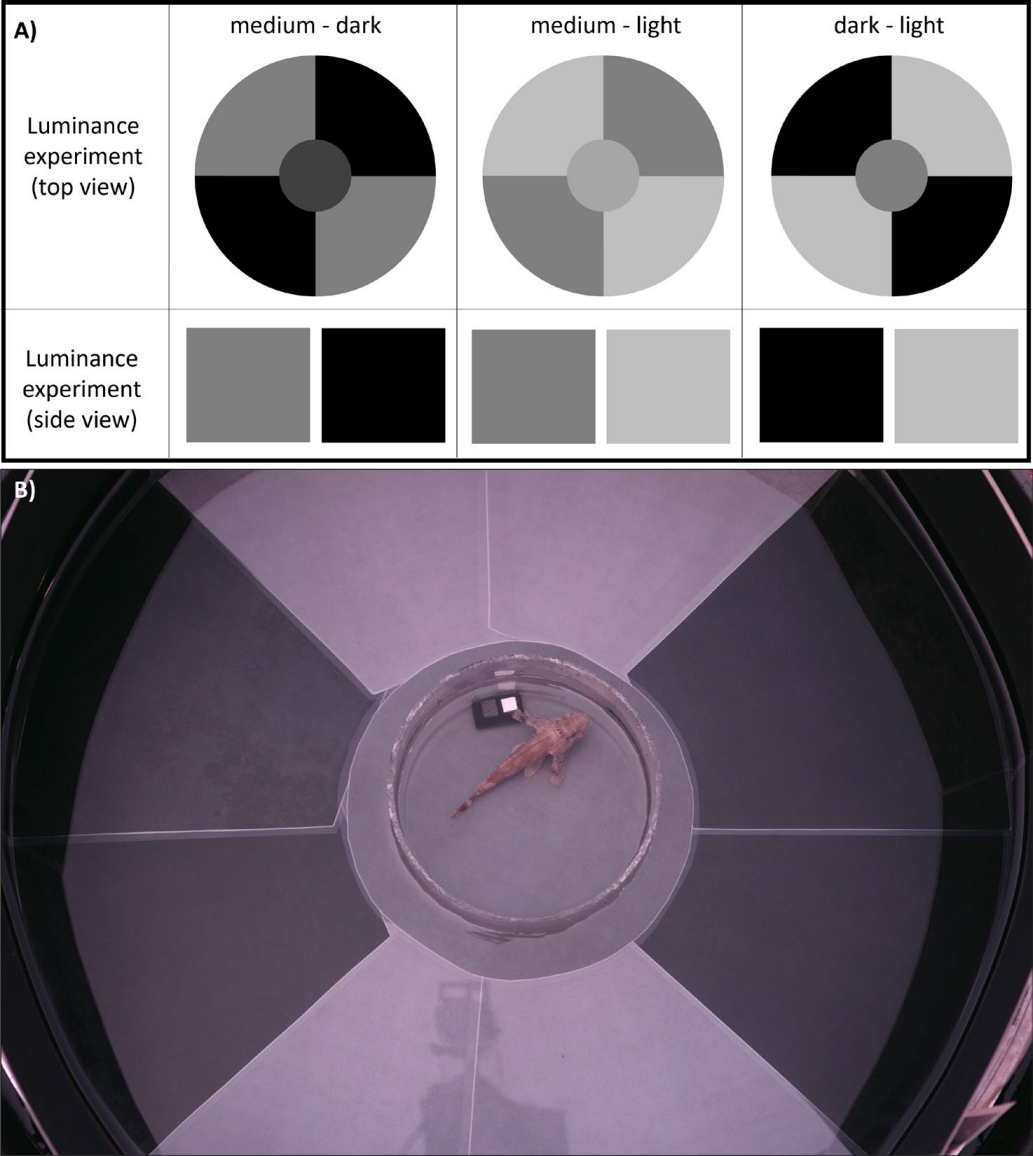
(A) Schematic view of the three choice treatments in the behavioural experiment. The circular area in the centre represents the starting zone. (B) Exemplary photo of the setup, choice treatment ‘medium‐dark’. The scorpionfish is in the acclimation cylinder without the opaque walls inserted, so it can see the setup before being released.

**FIGURE 2:**
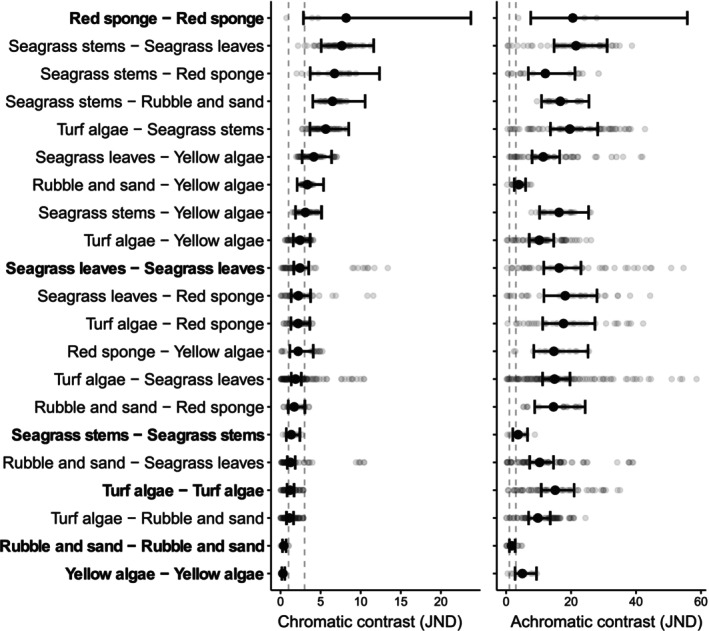
Chromatic and achromatic contrast between and within substrate type as perceived by scorpionfish. For each substrate comparison, grey points represent individual calculations of Just Noticeable Difference (JND) based on average cone catches measures from each photo of the first substrate type against the same from each photo of the second substrate. Markers with vertical bars represent predicted medians and 95% compatibility intervals (CIs) derived from 10,000 simulations of the posterior distribution of model parameters (*R* ^2^_cond_ = 0.670, *R* ^2^_marg_ = 0.436 for chromatic and *R* ^2^_cond_ = 0.443, *R* ^2^_marg_ = 0.258 for achromatic contrast model). The dashed lines indicate one and three JND. Bold labels indicate within substate type comparisons.

We used three identical arenas made from plant pot saucers (60 cm diameter and 9 cm height, Primavera 70, Plastkon product s.r.o.) with inserted plastic cylinders (60 cm diameter and 21 cm height) as side walls. The bottom and walls were covered with printed and laminated paper (matte laminating pouches 125 μm, no. S‐PP525‐22, PRT GmbH). Backgrounds were printed by WiesingerMedia GmbH (Tübingen). The setup was split into quadrants, which were alternatingly covered with two backgrounds (Figure 1), to minimise possible orientation preference effects. The centre of the setup was starting zone (18 cm diameter disk) of intermediate luminance to the used backgrounds. At the beginning of a trial, an acclimation cylinder (15 cm diameter, 10 cm height) of transparent plexiglass was placed in the starting zone. For the acclimation time, the cylinder had opaque inner walls of the same colour and luminance as the starting zone. Attached inside the cylinder were two grey standards (12% and 72%) needed for calibrated photography (Figures 1B and 2B). Trials were filmed with a GoPro Hero 7, and RAW photographs were taken at the beginning and the end of trials with a calibrated Nikon D4 DSLR camera (Nikkor 60 mm macro lens). The camera was placed on a tripod at ~100 cm distance from the setup, looking down at a ~20° angle. The GoPro was mounted on top of the camera, looking down at approximately the same distance and angle. Setups were filled with fresh seawater before each trial (water level 9 cm). At the start of each trial, a scorpionfish was caught from a housing tank using hand nets and transferred to the setup in a white bucket. After that, the GoPro recording was started, and the scorpionfish was gently transferred into the acclimation cylinder. Scorpionfish were allowed to acclimate for 1 min. Then, the opaque walls of the acclimation cylinder were lifted, allowing the scorpionfish to see the rest of the setup for 1 min. Photos of the fish were taken before and after this 1 min of acclimation. The acclimation cylinder was then lifted, and the scorpionfish were filmed as they moved in the setup for another 10 min. Then, the GoPro recording was stopped, and scorpionfish were gently directed towards the center of the setup using the transparent acclimation cylinder. At this point, scorpionfish were photographed once more in the starting zone. Moving the scorpionfish to the starting zone and taking the last photo took < 5 s, which is too little time for the fish to change colour again (John et al. 2023). The scorpionfish were then placed back into the bucket with a hand net, either to be transferred to the next choice treatment or returned to the housing tank. The order of the three choice treatments was balanced across individuals of each species. Setup orientation was alternated and balanced across individuals for each choice treatment.

#### 2.2.2 Experimental Backgrounds

Scorpionfish were presented with three binary choices between all combinations of a *light*, *medium* and *dark grey* background (Figure 1A). To create the experimental backgrounds, we took standardised photos of a grey scale under experimental light conditions and calculated the average luminance of the grey values from the perspective of the scorpionfish ( *S. porcus* ) visual system (Govardovskii and Zueva 1988; John et al. 2023, 2024; Schweikert et al. 2018). We then calculated the Weber contrast of average scorpionfish body luminance when adjusted to a light, medium and dark grey background (data from: John et al. 2023; John et al. 2024) against each grey value of the scale. For the *medium* background, we chose the grey value that had the smallest Weber contrast (0.03) to the medium‐grey adapted scorpionfish. The *light* and *dark* backgrounds were instead made with grey values with the same absolute Weber contrast (0.33) to the light‐ or dark‐adapted scorpionfish. The central starting zone and the acclimation cylinder had an intermediate luminance between the respective two experimental backgrounds.

#### 2.2.3 Behavioural Data Analysis

**FIGURE A1:**
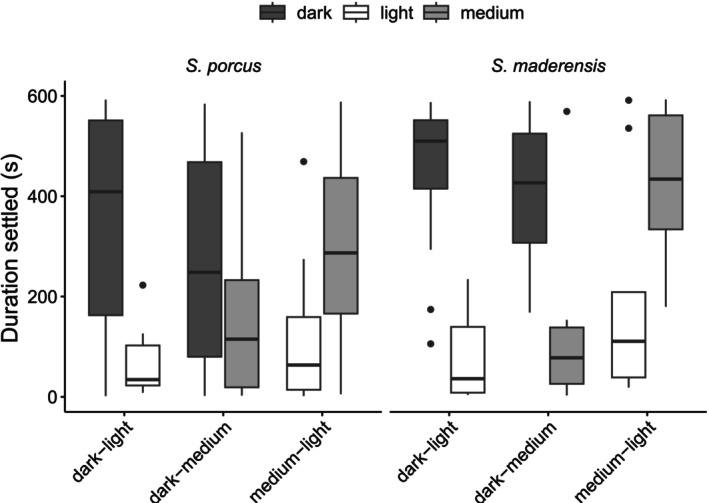
Absolute duration (seconds) that both scorpionfish species were settled on both backgrounds in each choice treatment of the behavioural experiment split by background combination (*x*‐axis) and background (colour).

To analyse background preferences from the videos, we used the program BORIS (version 8.20.4) (Friard and Gamba 2016). We measured how much time an individual settled on each background. Initial observations showed that scorpionfish sometimes display a ‘stop‐and‐go’ movement, where they would pause for a few seconds but eventually move on, which we did not consider as a choice. To exclude these occasions, we only included events where scorpionfish did not move for at least 5 s (*duration settled*). If a scorpionfish was sitting on both backgrounds, we considered the background under its eyes as the one chosen. We also noted when an individual settled for the first time on a background without moving for at least 1 min (choice variable *first settled*). Because *duration settled* showed the same pattern as *first settled* (Figure A1 in Appendix A), we only display results for *first settled*.

### 2.3 Body Colouration on Chosen Background

To analyze scorpionfish colouration in relation to background choice, we converted the scorpionfish images taken after each trial to cone catches (see procedure described above in Section 2) of the natural prey fish species *Tripterygion delaisi* (Santon et al. 2020, 2021), which has cone sensitivities peaking at 468, 517 and 530 nm (Bitton et al. 2017) and a cone ratio of 0.25:1:1 (Fritsch et al. 2017). For *T. delaisi* , we assumed a Weber fraction of 0.05 for the most abundant cones and for the luminance channel (Champ et al. 2016; Olsson et al. 2018). The luminance channel was defined as the average cone catches of the two longer wavelength sensitive cones (John et al. 2023; Lythgoe 1979). Our photography and model illuminant were a D65 spectrum (standard daylight) because this was the spectrum under which the behavioural experiment took place. Both scorpionfish species have a body pattern of alternating dark and light bars, so we selected two patches within two of those bars that are typically well distinguishable, located close to the tail fin, as regions of interest (ROI). We then measured cone catches for these bars (variable 1: *luminance channel cone catches* for dark and light bar). We also selected eight 1 cm^2^ samples per background from randomly selected photos and measured their cone catches to calculate the achromatic contrast of the two bars against the background that each scorpionfish chose in each trial (for details on contrast calculation see Section 2) (variable 2: *achromatic background contrast* of dark and light bar against the chosen background). We moreover calculated the achromatic contrast of both bars within one individual's pattern as an estimate of internal pattern contrast (variable 3: *internal pattern contrast* between dark and light bar).

### 2.4 Statistical Analysis

Following a guided linear modelling routine for R (Santon et al. 2023), we used the glmmTMB package (Brooks et al. 2017) to implement generalised linear mixed models and analyze (1) chromatic and achromatic contrast between and within natural substrate types, (2) choice in the behavioural experiment, and (3) scorpionfish body colouration parameters on the chosen background. The routine guides through model assessment by inspection of the distribution of randomised quantile residuals, computed with the R‐package DHARMa (Hartig 2022), within and among factor predictor levels that were included or not in the models, and by performing posterior predictive checks to assess model dispersion and overall model fit. We chose the family distributions based on the nature of each response variable used. We use the *r*2 function of the performance package (Lüdecke et al. 2021) to report marginal and conditional *R* ^2^ as a measure of fit for each model (Nakagawa and Schielzeth 2013). We graphically report model predicted medians and their 95% compatibility intervals (CIs) calculated from the posterior distributions of fitted values obtained from 10,000 sets of model parameters (Brooks et al. 2017).

### 2.5 Visual Contrasts of Natural Backgrounds

We calculated contrasts between and within the six substrate types, making a total of 21 comparisons. Our sample size per substrate was *n* = 12 for *rubble and sand*, *n* = 9 for *seagrass leaves*, *n* = 9 for *turf algae*, *n* = 3 for *red sponge*, *n* = 5 for *yellow algae* and *n* = 5 for *seagrass stems*. We implemented two models using a Gamma distribution (link = log) for the response variables *achromatic contrast* and *chromatic contrast*. We specified *substrate comparison* as a fixed effect and *photo ID* as a random factor.

### 2.6 Background Choice

From the initial 23 *S. maderensis* and 27 *S. porcus* , five *S. porcus* individuals never settled on any experimental background and were therefore excluded from analysis. We re‐encoded the variable *first settled* into a hypothesis‐driven success/failure variable that indicated whether an individual first settled on the background we expected it to settle (1), or on the opposite background (0) (Table 1).

**TABLE 1:** Expectations of scorpionfish preference to settle on one of the backgrounds in each choice treatment.

Choice treatment	Success/Expected (1)	Failure/Unexpected (0)
Dark–light	Dark	Light
Medium–light	Medium	Light
Medium–dark	Medium	Dark

We then implemented a model using a binomial distribution (link = logit) for the response variable *first settled (success/failure)*, with scorpionfish *species* and *choice treatment* and their interaction as fixed effects. We specified *fish ID* as a random factor. To report results, we extracted from the model the median proportion of expected choice for each treatment from 10,000 simulations of the joint posterior distribution of model parameters and their 95% compatibility intervals (CIs). CIs overlapping the random choice threshold of 0.5 (50% chance of fish to choose either background randomly as they had an equal area per treatment) indicate a random choice (Newport et al. 2024).

### 2.7 Body Colouration on Chosen Background

To understand how different bars in the scorpionfish body colouration contribute to camouflage, we analyzed *luminance channel cone catches* of dark and light bars and *achromatic background contrast* of dark and light bars against the backgrounds that fish chose in each choice treatment, and *internal pattern contrast* of individuals on the chosen background. We excluded cases where individuals chose the light background because this rarely occurred (*n* = 5 for *S. maderensis* and *n* = 7 for *S. porcus* ). This left a total of 41 preferences for the dark background by *S. maderensis* and 34 by *S. porcus* , and 18 preferences for the medium background by *S. maderensis* and 20 by *S. porcus* . We implemented models using a Gamma distribution (link = log) for the response variables *luminance channel cone catches*, *achromatic background contrast* and *internal pattern contrast*. We specified scorpionfish *species*, *bar* and *chosen background* and their interaction as fixed effects. We used *fish ID* as a random factor.

## 3 Results

### 3.1 Visual Contrasts of Natural Backgrounds

Chromatic contrast was generally lower than achromatic contrast between the different substrate types from the scorpionfish perspective. Chromatic contrast was below the detection threshold of one JND only for within‐substrate comparisons of *rubble and sand* and *yellow algae*. However, several comparisons were below the more conservative detection threshold of three JND (Figure 2; Table A1 in Appendix A), indicating a low chromatic diversity within different patches of the same substrate as well as between different substrates. Achromatic contrast was generally above three JND (Figure 2). This did not only apply to comparisons between different substrate types, but within samples of the same substrate (Figure 2).

### 3.2 Background Choice

**FIGURE 3:**
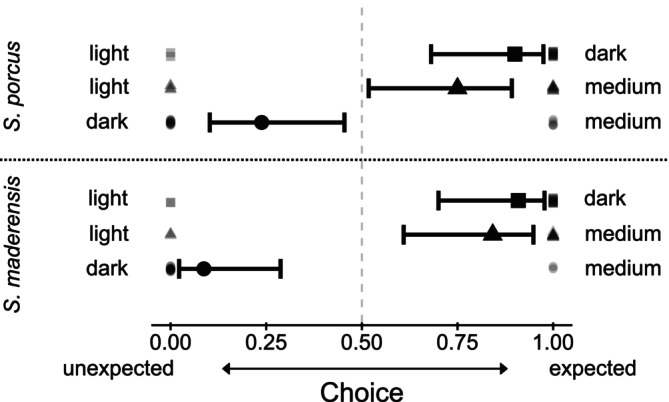
Choice of *S. maderensis* and *S. porcus* for all three background combinations in the behavioural experiment. Choice was defined by a scorpionfish settling for at least 1 min for the first time. Points represent choice of each individual (*n* = 23 *S. maderensis* , *n* = 22 *S. porcus* , note that not every individual settled in every choice treatment) and were scored with 1 for a choice that was expected by our hypothesis and with 0 for a choice against our hypothesis. Markers with horizontal bars represent predicted medians and 95% compatibility intervals (CIs) derived from 10,000 simulations of the posterior distribution of model parameters. *R* ^2^_cond_ = 0.499. The dashed line indicates the random choice threshold 0.5. CIs excluding 0.5 indicate a non‐random choice.

Both species showed the same preferences (Figure 3). On the light versus dark background, scorpionfish chose the dark background according to our predictions (choice estimate and CIs for *S. maderensis* : 0.91 [0.70–0.98], *S. porcus* : 0.90 [0.68–0.97]). On the light versus medium background, scorpionfish chose the medium background according to our predictions (choice estimate and CIs for *S. maderensis* : 0.84 [0.61–0.95], *S. porcus* : 0.75 [0.52–0.89]). On the dark versus medium background, scorpionfish chose the dark background against our predictions (choice estimate and CIs for *S. maderensis* : 0.09 [0.02–0.29], *S. porcus* : 0.24 [0.10–0.45]). For all background comparisons, scorpionfish made a non‐random choice (i.e., CIs of choice estimates never overlap 0.5).

### 3.3 Body Colouration on Chosen Background

**FIGURE 4:**
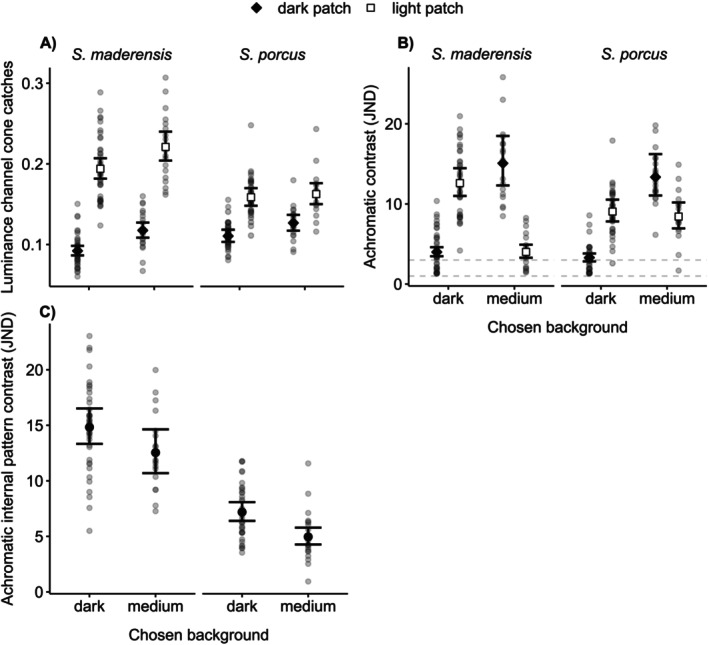
Body colouration of *S. maderensis* and *S. porcus* after choosing a background in the behavioural experiment as perceived by the prey fish *Tripterygion delaisi* . (A) Luminance channel cone catches of the two dark and light bars, and achromatic contrast expressed as Just Noticeable Differences (JND) of the two bars (B) against the chosen background and (C) against each other. The dashed lines (B) indicate one and three JND. Colour and shape of the markers indicate the dark or light bar (A, B). Grey points represent individual cone catches (A) or JND (B, C). Markers with vertical bars represent predicted medians and 95% compatibility intervals (CIs) derived from 10,000 simulations of the posterior distribution of model parameters. Cases where the light background was chosen were excluded because of the low number of individuals that showed this preference (*n* = 23 *S. maderensis* , *n* = 22 *S. porcus* tested in three choice treatments).

*S. maderensis* individuals decreased the luminance of both their dark and light bars when choosing the dark over the medium background (Figure 4A, Table 2A). *S. porcus* individuals only decreased the luminance of their dark bars (Figure 4A, Table 2A). When calculating achromatic contrast of the dark and light bars of each fish against the background chosen, the lowest achromatic contrast was found for the dark bars on the dark background and for the light bars on the medium background (Figure 4B, Table 2B). *S. maderensis* had similarly high internal contrast regardless of their chosen background, while *S. porcus* reduced internal contrast when choosing the medium compared to the dark background (Figure 4C, Table 2C). Compared to *S. porcus, S. maderensis* expressed higher internal contrast on both medium and dark backgrounds (Figure 4, Table 2C).

**TABLE 2:** Pairwise contrasts of light or dark bar metrics of *S. maderensis* and *S. porcus* as perceived by *Tripterygion delaisi* .

Comparison	Ratio	CIs	Ratio	CIs
(A) Luminance channel cone catches of bars on chosen backgrounds (*R* ^2^_cond_ = 0.847, *R* ^2^_marg_ = 0.722)				
	*S. maderensis*		*S. porcus*	
Dark bar on medium vs. dark background	**0.78**	**0.73, 0.85**	**0.87**	**0.81, 0.94**
Light bar on medium dark background	**0.88**	**0.81, 0.95**	0.98	0.90, 1.05
(B) Achromatic contrast of bars to chosen backgrounds (*R* ^2^_cond_ = 0.647, *R* ^2^_marg_ = 0.632)				
	*S. maderensis*		*S. porcus*	
Dark bar to medium vs. dark background	**0.26**	**0.21, 0.34**	**0.25**	**0.19, 0.31**
Light bar to medium dark background	**3.15**	**2.48, 4.00**	1.07	0.85, 1.36
(C) Achromatic internal pattern contrast (*R* ^2^_cond_ = 0.647, *R* ^2^_marg_ = 0.616)				
	*S. maderensis*		*S. porcus*	
Contrast on medium vs. dark background	1.18	0.98, 1.42	**1.45**	**1.21, 1.74**
	Dark background chosen		Medium background chosen	
*S. porcus* vs. *S. maderensis*	**2.06**	**1.76, 2.42**	**2.53**	**2.03, 3.14**

*Note:* Contrasts expressed as response ratios of (A) luminance channel cone catches of the same bar on the chosen backgrounds for each species, and (B) achromatic contrasts of the same bar to chosen backgrounds for each species and (C) achromatic internal pattern contrast between the two backgrounds for each species, and between the two species for the same background chosen. Effect size is proportional to the deviation of ratios from one, and the robustness of the result increases with decreasing degree of overlap of the 95% compatibility intervals (CIs) with one. Response ratios with CIs excluding one are highlighted in bold. *N* = 23 for *S. maderensis* and *N* = 22 for *S. porcus* .

## 4 Discussion

### 4.1 Visual Contrasts of Natural Backgrounds

Only a few natural substrates had a chromatic contrast below one Just Noticeable Difference (JND) and can therefore be considered indistinguishable for scorpionfish in the modelled depth of 6 m in mid‐day light conditions. However, under natural viewing conditions, using a more conservative perception thresholds such as three JND to interpret results (Abernathy et al. 2017; Siddiqi et al. 2004; da Silva et al. 2020; Stevens et al. 2014), most substrate types appeared similar. In an environment where only a few substrates clearly differ from others, a generalist body colouration might be promoted (Merilaita et al. 1999). Here, animals typically match no or only a few backgrounds perfectly, but show a sub‐optimal match to many backgrounds (Hughes et al. 2019). This could mitigate the need for other camouflage strategies such as background choice. However, looking at average achromatic contrast, substrate types were markedly different from each other, with certain cases also showing high contrast variability within samples of the same substrate type. This reflects the importance that perceived luminance may have for scorpionfish to assess their environment and is possibly the reason why dynamic luminance changes are much stronger than hue changes in these fishes (John et al. 2023). It also indicated that achromatic cues might be more important than chromatic cues as factors driving a potential background choice in scorpionfish.

### 4.2 Background Choice

In the choice experiment, scorpionfish preferentially settled on the darker background in all treatments. When choosing between a background of perceived luminance within their own colouration range (‘medium’) and a darker background, scorpionfish preferred the darker alternative. The data show that scorpionfish actively choose between backgrounds, but they chose against our predictions. A preference for dark backgrounds was previously shown in several fish species (Bradner and McRobert 2001; Kjernsmo and Merilaita 2012; Smithers et al. 2018) and might indicate an escape response. Individuals may feel threatened in the novel environmental setup and consequently choose a background that suggests shelter in their natural habitat, that is, dark cracks or crevices. This does not exclude that in a foraging context under natural conditions, scorpionfish would instead prefer to settle on backgrounds that are more similar to their own luminance and therefore enhance background matching.

We used average scorpionfish luminance to predict their choice according to the strategy of background matching, even though scorpionfish have a contrasting body pattern dominated by bars and patches. Calculating the background match of individual bars within the scorpionfish pattern revealed that their dark bars had the lowest achromatic contrast on the dark background. Therefore, scorpionfish may have preferred this background to improve camouflage through disruptive colouration. Here, the different bars could allow differential blending, and in *S. porcus* , the increased internal contrast between light and dark bars indicates aiming for maximum disruptive contrast (Stevens and Merilaita 2009b). Disruptive colouration is a more generalist camouflage strategy compared to background matching, because it offers crypsis on a wider range of backgrounds (Cuthill et al. 2005; Phillips et al. 2017; Price et al. 2019; Robledo‐Ospina et al. 2017). Therefore, scorpionfish could show a preference for backgrounds that do not match their average appearance best but rather disrupt their body outline the most.

### 4.3 Species Comparison

We tested two congeneric scorpionfish species to understand potential differences and similarities in their body colouration and behaviour. *S. maderensis* is a species known to be common around eastern Atlantic islands and only in specific areas in the Mediterranean, predominantly the southern Mediterranean (La Mesa et al. 2005), while *S. porcus* is common all over the Mediterranean (Fricke et al. 2018). In the light of a progressively changing environment, understanding a species' potential to outcompete congeneric species is of great interest. Since both species are competing for the same habitat and share the same lifestyle, differences in camouflage strategies could indicate relevant niche partitioning (Stevens and Ruxton 2019). Previous studies indicated a greater individual variation in dominant pattern marking size for *S. porcus* (John et al. 2024), and that *S. maderensis* has an overall more light colouration (John et al. 2023). The present results show that *S. maderensis* achieves a higher internal pattern contrast compared to *S. porcus* . While *S. porcus* reduces their internal pattern contrast when settling on the medium background, *S. maderensis* maintains a high contrast even in that case. This could indicate a differential strategy where *S. porcus* aims for background matching when choosing the medium background, while *S. maderensis* remains with a maximum contrast, disruptive strategy. This is supported by the fact that *S. maderensis* is more likely to achieve differential blending even on the medium background, as indicated by a low contrast of the light bars to the medium background. This might not be possible for the overall darker *S. porcus* (John et al. 2023), and therefore a background matching strategy could be more beneficial when choosing the medium background. However, overall, here we find similar results for background choice for both species. We cannot exclude that this would be different under more natural conditions, where also other factors such as shapes, texture and chemical cues can add to background choice, which would more accurately reflect possible differences in microhabitat choice.

## 5 Conclusion

Overall, we showed that achromatic cues should drive background choice in scorpionfish, while chromatic cues should not be of great relevance. We also showed that scorpionfish do actively choose between different backgrounds, preferring darker ones. We cannot exclude that the choice for dark backgrounds we observed in our experiments is driven by an escape response. However, assuming fish chose to improve their camouflage, it seems more likely that scorpionfish enhance their camouflage via disruptive colouration by choosing a specific background. How the two congeneric scorpionfish species use camouflage strategies in different natural microhabitats and understanding potential differences between species should be investigated in future studies.

## Author Contributions

**Leonie John:** conceptualization (equal), data curation (lead), formal analysis (lead), investigation (lead), methodology (lead), visualization (lead), writing – original draft (lead), writing – review and editing (equal). **Matteo Santon:** conceptualization (equal), formal analysis (supporting), methodology (supporting), writing – review and editing (equal). **Nico K. Michiels:** conceptualization (equal), methodology (supporting), writing – review and editing (equal).

## Conflicts of Interest

The authors declare no conflicts of interest.

## Data Availability

All data collected and analyzed in the study will be available on Figshare, doi: https://doi.org/10.6084/m9.figshare.29045450.

## Appendix A

**TABLE A1:** Chromatic and achromatic contrast of substrate types against each other as perceived by *S. porcus* .

Substrate type comparison	Chromatic contrast		Achromatic contrast	
	Estimate	CIs	Estimate	CIs
Red sponge–Red sponge	8.12	2.85, 23.74	20.26	7.66, 55.83
Seagrass stems–Seagrass leaves	7.69	5.07, 11.62	21.43	14.87, 31.14
Seagrass stems–Red sponge	6.74	3.70, 12.35	12.09	6.85, 21.00
Seagrass stems–Rubble and sand	6.49	4.03, 10.55	16.64	10.83, 25.31
Turf algae–Seagrass stems	5.62	3.67, 8.51	19.58	13.67, 27.97
Seagrass leaves–Yellow algae	4.16	2.70, 6.37	11.46	7.93, 16.54
Rubble and sand–Yellow algae	3.34	2.09, 5.37	3.90	2.53, 5.97
Seagrass stems–Yellow algae	3.12	1.88, 5.12	16.32	10.37, 25.45
Turf algae–Yellow algae	2.43	1.59, 3.70	10.23	7.20, 14.63
Seagrass leaves–Seagrass leaves	2.43	1.67, 3.51	16.34	11.60, 23.16
Seagrass leaves–Red sponge	2.22	1.32, 3.74	18.11	11.62, 28.39
Turf algae–Red sponge	2.17	1.30, 3.66	17.61	11.22, 27.28
Red sponge–Yellow algae	2.19	1.16, 4.08	14.67	8.51, 25.36
Turf algae–Seagrass leaves	**1.87**	**1.35, 2.60**	14.85	11.21, 19.77
Rubble and sand–Red sponge	1.69	0.97, 3.05	14.57	8.72, 24.35
Seagrass stems–Seagrass stems	**1.34**	**0.74, 2.42**	3.68	2.06, 6.65
Rubble and sand–Seagrass leaves	**1.25**	**0.84, 1.84**	10.32	7.28, 14.65
Turf algae–Turf algae	**1.18**	**0.81, 1.70**	15.03	10.82, 20.92
Turf algae–Rubble and sand	**1.09**	**0.75, 1.62**	9.73	7.01, 13.64
Rubble and sand–Rubble and sand	***0.41***	***0.24, 0.69***	**1.70**	**1.01, 2.79**
Yellow algae–Yellow algae	***0.29***	***0.16, 0.54***	4.99	2.75, 9.37

*Note:* For each substrate comparison, the estimate is the predicted median of Just Noticeable Difference (JND) based on cone catches from each photo of the first substrate type against each photo of the second substrate. Estimates and 95% compatibility intervals (CIs) were derived from 10,000 simulations of the posterior distribution of model parameters (*R* ^2^_cond_ = 0.670, *R* ^2^_marg_ = 0.436 for chromatic and *R* ^2^_cond_ = 0.443, *R* ^2^_marg_ = 0.258 for achromatic contrast model). Estimates with CIs below three JND are highlighted in bold; estimates with CIs below one are highlighted in italic.
